# An unusual ECG change after a car crash

**DOI:** 10.1111/anec.12835

**Published:** 2021-02-19

**Authors:** Yi Li, Tong Liu, Yajuan Shi, Xiaolin Shi

**Affiliations:** ^1^ Department of Cardiothoracic Surgery Wuhan Asia Heart Hospital Affiliated to Wuhan University of Science and Technology Hubei China; ^2^ Tianjin Key Laboratory of Ionic‐Molecular Function of Cardiovascular Disease Department of Cardiology Tianjin Institute of Cardiology Second Hospital of Tianjin Medical University Tianjin China; ^3^ Department of Remote ECG Diagnosis Center Qidong People's Hospital (Qidong Hospital Affiliated to Nantong University) Jiangsu China

**Keywords:** abnormal repolarization, Brugada phenocopy, chest trauma, dynamic changes

## Abstract

A 34‐year‐old male patient was preparing for splenic artery embolization because of a car crash. Personal or family histories of cardiovascular diseases, sudden cardiac death, or Brugada syndrome were denied. Type 1 Brugada pattern was observed in the preoperative electrocardiogram and gradually resolved within a week. Chest blunt trauma may contribute to the transient ECG changes, and some particular considerations should be taken in this patient.

## 1 CASE PRESENTATION

**FIGURE 1:**
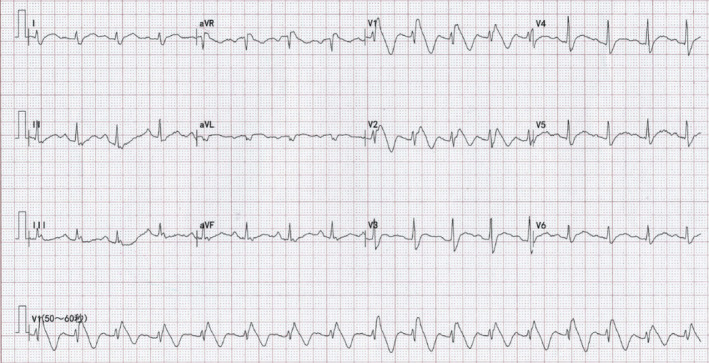
ECG recorded in the emergency room

**FIGURE 2:**
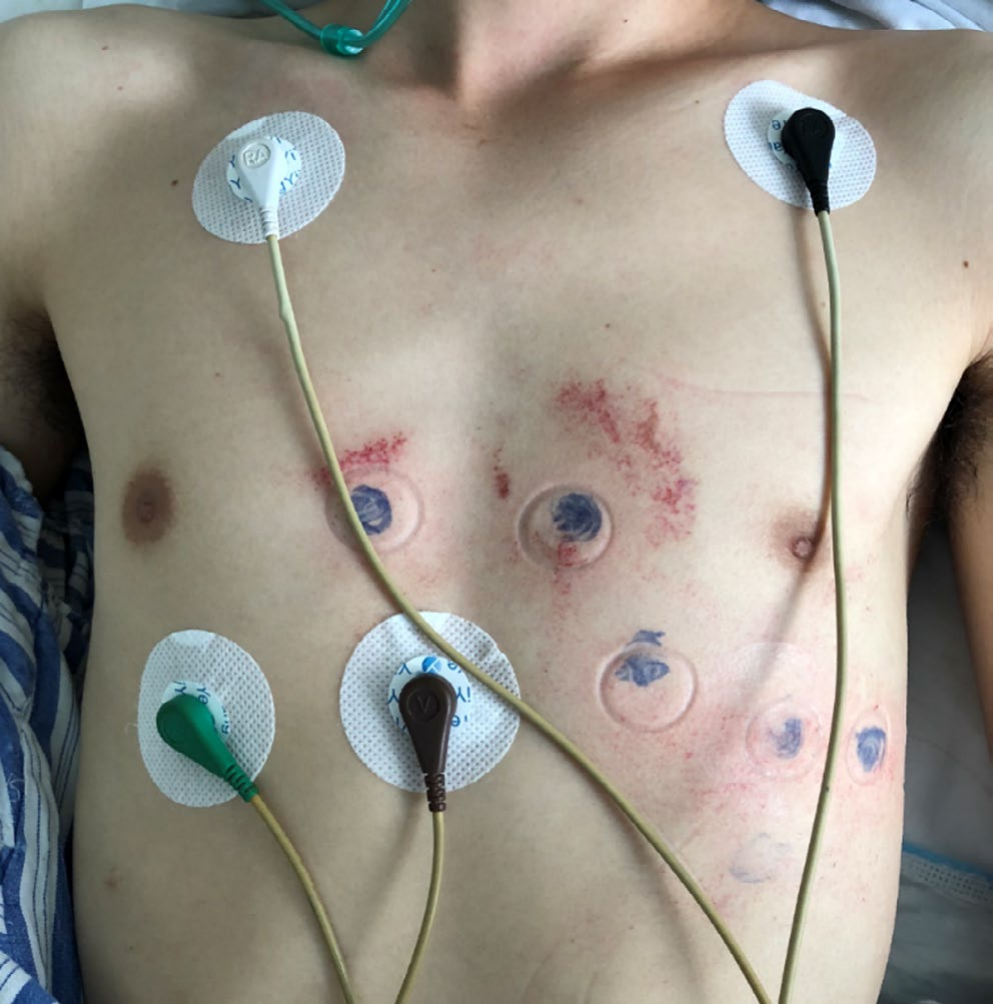
Chest trauma location and precordial electrodes markers

**FIGURE 3:**
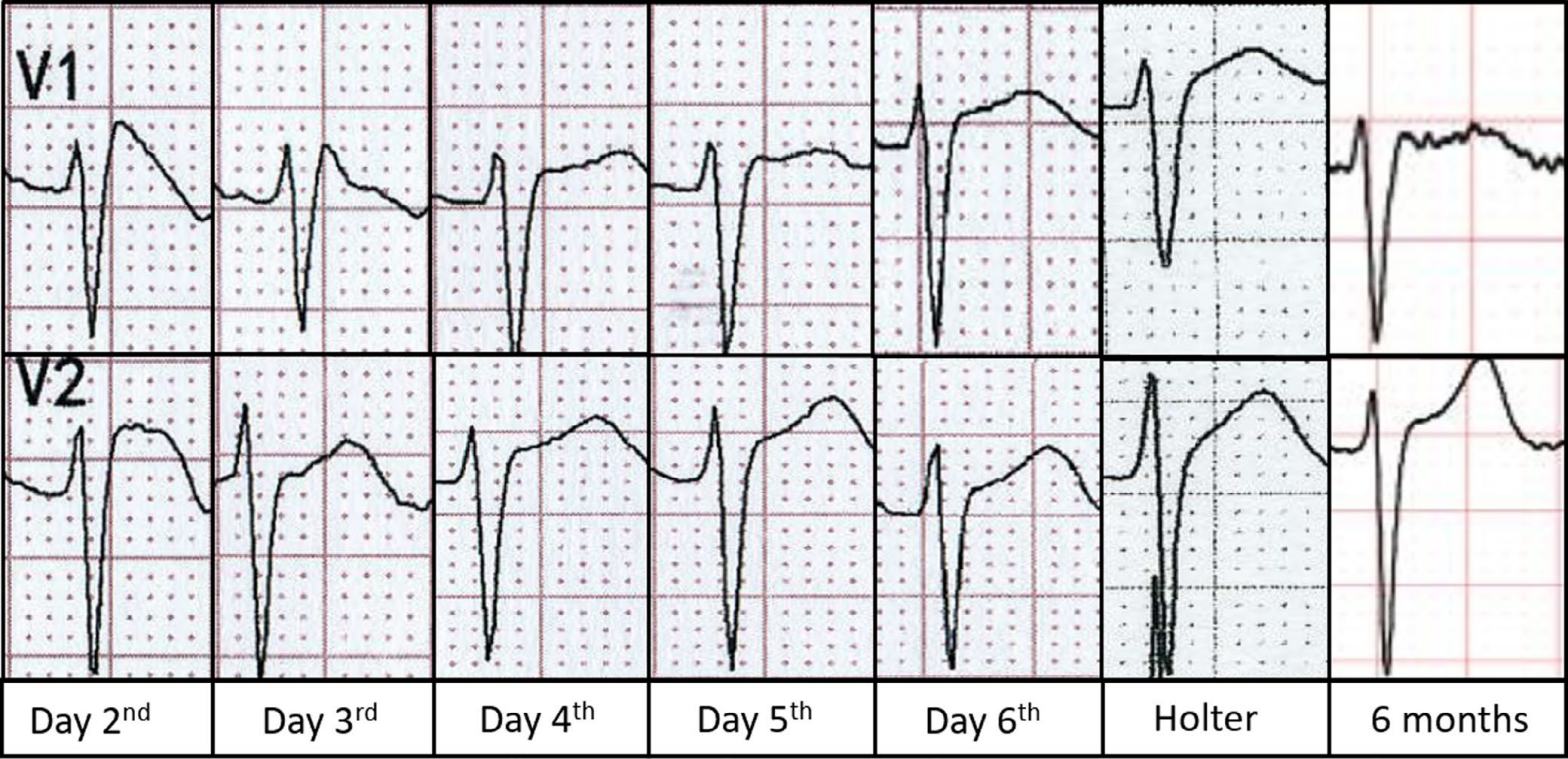
Dynamic changes of ECGs during admission

A 34‐year‐old male patient was admitted to the emergency room with left abdominal pain following a car accident. Physical examination indicated stable vital signs and no obvious cardiac abnormalities. Abdominal ultrasound showed perisplenic effusion (15 mm), while echocardiography showed no abnormality. His troponin T (0.068 ng/ml), creatine kinase (171 U/L), and creatine kinase–MB (99 U/L) were mildly elevated, and electrolyte levels were normal. History of previous medication and cardiovascular disease was denied. Before he underwent emergency splenic artery embolization for splenic contusion, a routine 12‐lead ECG was performed (Figure 1), which showed sinus rhythm (99 bpm) with QRS complex widening in terminal, and an ST segment elevated ≥ 2 mm followed by a symmetric negative T wave in V1‐V2 (type 1 Brugada pattern). More postoperative ECGs were reexamined at the same electrodes position (Figure 2), and Brugada pattern gradually resolved in next few days (Figure 3). Troponin T backed to normal within a week and three chest computed tomography (CT) scans indicated no abnormality in mediastinum during admission. No more Brugada pattern was found in the Holter test before discharge and reexamination 6 months later (Figure 3). Medications known to induce Brugada patterns were not prescribed. Provocative testing and genetic testing were declined.

## 2 DISCUSSION

The presentation and transient dynamic changes of ECGs in our case were compatible with the type 1 Brugada phenocopy (BrP), which are clinical entities that refer to ECG patterns identical to ECG presentations observed in Brugada syndrome **(**de Oliveira Neto et al., 2019 **)**. However, they may have different etiologies, such as metabolic conditions, mechanical compression, myocardial ischemia, pulmonary embolism, myocardial or pericardial disease, antiarrhythmic drug application, and others **(**Baranchuk et al., 2012; Zhang et al., 2017; Sreenivasan et al., 2018; Gottschalk et al., 2016 **)**. The potential reason for the BrP in our case may be associated with chest blunt trauma. The main area of chest trauma in this patient was located in the precordial region at the left margin of the sternum, which was just the surface projection of the right ventricular outflow tract (RVOT). Pérez‐Riera et al. (2018) reported a case of BrP induced by mediastinal tumor mechanical compression on the RVOT without elucidated the mechanism. Mechano‐electrical feedback caused by acute blunt impact to the ROVT region may lead to the abnormality in ventricular repolarization **(**Link, 2012), which may contribute to this transient BrP pattern.

Compared to the patients with single chest/abdominal trauma, following particular considerations should be taken in the patients with the Brugada pattern. First, commotio cordis caused by sudden blunt external force to the precordium should be alerted. Although this patient did not develop malignant ventricular arrhythmia during admission, the transient repolarization abnormality indicated by the Brugada pattern may be a potential danger signal. Therefore, ECG examination should be carried out in time for such patients with chest trauma to avoid adverse cardiac events during admission. Furthermore, it was imperative to identify the incentive of BrP. For the patients with severe chest blunt trauma, compression of mediastinal hematoma and pericardial effusion were not uncommon, which may contribute to cause the BrP **(**de Oliveira Neto et al., 2019 **)**. Chest CT scan and echocardiography are helpful for early detection of these abnormalities. The last but not the least, general anesthesia (GA) is usually performed preoperatively in patients with severe trauma; however, the safety of GA in patients with Brugada pattern is remain controversial **(**Ciconte et al., 2018 **)**. Increased vagal tone after GA could induce bradycardia, which may lead to the Brugada wave more pronounced. Large doses of anesthetics such as propofol may evoke the Brugada pattern by affecting ion channels in the RVOT myocardium.

It is worth mentioning that the patient refused the further examinations, though the genetic test (not mandatory) and provocative test were pivotal to the differential diagnosis of BrP (Gottschalk, et al., 2016). In consideration of the potential cardiac injury or arrhythmias, we did not force the patient to undergo provocative testing with sodium channel blockers, which may be the limitation of this case.

## 3 CONCLUSION

We presented a case of transient and self‐normalized BrP following car crash and chest blunt trauma may be a contributory inducement. For the patients with repolarization abnormality, extra cautions should be exercised in many aspects such as the use of drugs with potential influence on cardiac ion channels because the surgeons are inexperience in cardiac evaluation. Consulting a cardiologist timely may avoid the adverse cardiac events.

## CONFLICT OF INTEREST

The authors declared that they have no conflicts of interest to this work. We declare that we do not have any commercial or associative interest that represents a conflict of interest in connection with the work submitted.

## AUTHOR CONTRIBUTIONS

Conceptualized the Study, provided software and resources, performed formal analysis, and wrote the original draft: Yi Li. Designed methodology, and wrote and revised the manuscript: Tong Liu. Investigated the study, visualized the data, and administered the project: Yajuan Shi. Validated and curated data: Xiaolin Shi.

## ETHICAL APPROVAL

The study complied with the edicts of the Declaration of Helsink (World Medical Association, 2013) and was approved by the patient and his family. Given that this is a retrospective case report, informed consent was waived.
